# Sequential immunizations confer cross-protection against variants of SARS-CoV-2, including Omicron in *Rhesus macaques*

**DOI:** 10.1038/s41392-022-00979-z

**Published:** 2022-04-18

**Authors:** Wei Deng, Qi Lv, Fengdi Li, Jiangning Liu, Zhiqi Song, Feifei Qi, Qiang Wei, Pin Yu, Mingya Liu, Shasha Zhou, Yaqing Zhang, Hong Gao, Nan Wang, Zijing Jia, Kai Gao, Jiayi Liu, Chong Xiao, Haiquan Shang, Xiangxi Wang, Linlin Bao, Chuan Qin

**Affiliations:** 1 Beijing Key Laboratory for Animal Models of Emerging and Remerging Infectious Diseases, NHC Key Laboratory of Human Disease Comparative Medicine, Institute of Laboratory Animal Science, Chinese Academy of Medical Sciences and Comparative Medicine Center, Peking Union Medical College, Beijing, 100021 China; 2 National Center of Technology Innovation for Animal Model, Beijing, China; 3 CAS Key Laboratory of Infection and Immunity, National Laboratory of Macromolecules, Institute of Biophysics, Chinese Academy of Sciences, Beijing, 100101 China; 4 Department of Radiology, Beijing Anzhen Hospital, Capital Medical University, Beijing, China

**Keywords:** Vaccines, Infection

## Abstract

Variants of concern (VOCs) like Delta and Omicron, harbor a high number of mutations, which aid these viruses in escaping a majority of known SARS-CoV-2 neutralizing antibodies (NAbs). In this study, *Rhesus macaques* immunized with 2-dose inactivated vaccines (Coronavac) were boosted with an additional dose of homologous vaccine or an RBD-subunit vaccine, or a bivalent inactivated vaccine (Beta and Delta) to determine the effectiveness of sequential immunization. The booster vaccination significantly enhanced the duration and levels of neutralizing antibody titers against wild-type, Beta, Delta, and Omicron. Animals administered with an indicated booster dose and subsequently challenged with Delta or Omicron variants showed markedly reduced viral loads and improved histopathological profiles compared to control animals, indicating that sequential immunization could protect primates against Omicron. These results suggest that sequential immunization of inactivated vaccines or polyvalent vaccines could be a potentially effective countermeasure against newly emerging variants.

## Introduction

With a high rate of transmission and the capability to evade the host’s immune system, the Delta variant of SARS-CoV-2 (B.1.617.2), which has 23 mutations in the Spike (S) protein compared to the first identified variants of concern (VOC, Alpha strain), has rapidly overtaken the previously circulating variants to become the dominant strain. More recently, the Delta variant has been superseded by another highly transmissible variant, Omicron (B.1.1.529) variant.^1–3^

Many current vaccine developers have reported that their vaccines can provide protection against the Delta variant but with a slightly reduced efficacy.^4–6^ However, the effectiveness of vaccines against the Omicron variant has come under increased scrutiny because there are fifteen mutations clustered in the RBD of this variant.^7^ These mutations aid the Omicron variant in escaping the vaccine-induced antibodies, causing breakthrough infections. During 2–4 weeks post BNT162B2 vaccination, the geometric mean titers (GMT) against to Omicron were approximately 30-fold decreased when compared to the wild-type strain,^8^ and the two-dose vaccine effectiveness of the mRNA-1273 against Omicron was declined quickly to 5.9% at >270 days.^9^ Moreover, the neutralization titer against Omicron was also reduced to 9.6 at 4–6 months post 2-dose inactivated vaccine immunization.^10^ The situation is further compounded with the waning immune responses elicited by either vaccination or natural infection, thereby leading to substantially reduced protective efficacy over time, particularly six months after the vaccination. There is a consensus on bolstering protection against newly emerged variants by administrating booster doses.^11,12^ With regards to this, strategies such as sequential immunization with vaccines as booster doses could be explored to further enhance the immune protection offered by vaccines.

It has been recently reported that the seroconversion rates of NAbs against Omicron were increased up to 95% (57/60) at the 1-month post the 3rd dose of CoronaVac.^7^ Besides, the level of NAbs elicited by prime-boost vaccination of BBIBP-CorV (another approved inactivated vaccine) followed by recombinant protein vaccine booster increased by fivefold compared to the 2-dose immunization.^10^ However, many aspects related to immunization that are not adequately addressed, including a lack of data from challenge trials on the effectiveness of vaccines, objective identification of most effective immunization strategies based on the duration of antibodies elicited under different immunization strategies, and cross-neutralization antibody profiles, could hamper efforts aimed at harnessing the benefits of sequential immunization.^13,14^ Given the fact that Omicron, with a dramatically increased infectivity and enhanced transmissibility, has replaced the Delta variant, there is also an ongoing debate about whether the immune responses can be fine-turned to the Omicron variant by boosting with a tweaked (Omicron-based) vaccine, which, of course, costs at least a couple of months to be well-evaluated. Fortunately, some key mutations identified in Omicron have already existed in Beta and Delta variants, which raises a possibility that a bivalent vaccine (inactivated β + δ) booster immunization strategy might confer a reasonable protective efficacy against currently circulating SARS-CoV-2 variants.

In this study, inactivated vaccine immunized *Rhesus macaques* were re-immunized with either inactivated or recombinant protein-based or bivalent inactivated vaccines, then the monkeys were challenged with live SARS-CoV-2 variants to determine the effectiveness of homologous and heterologous immunity.

## Results

### Cross-neutralizing antibodies elicited by sequential immunization are effective against WH09 and Delta strains

**Fig. 1:**
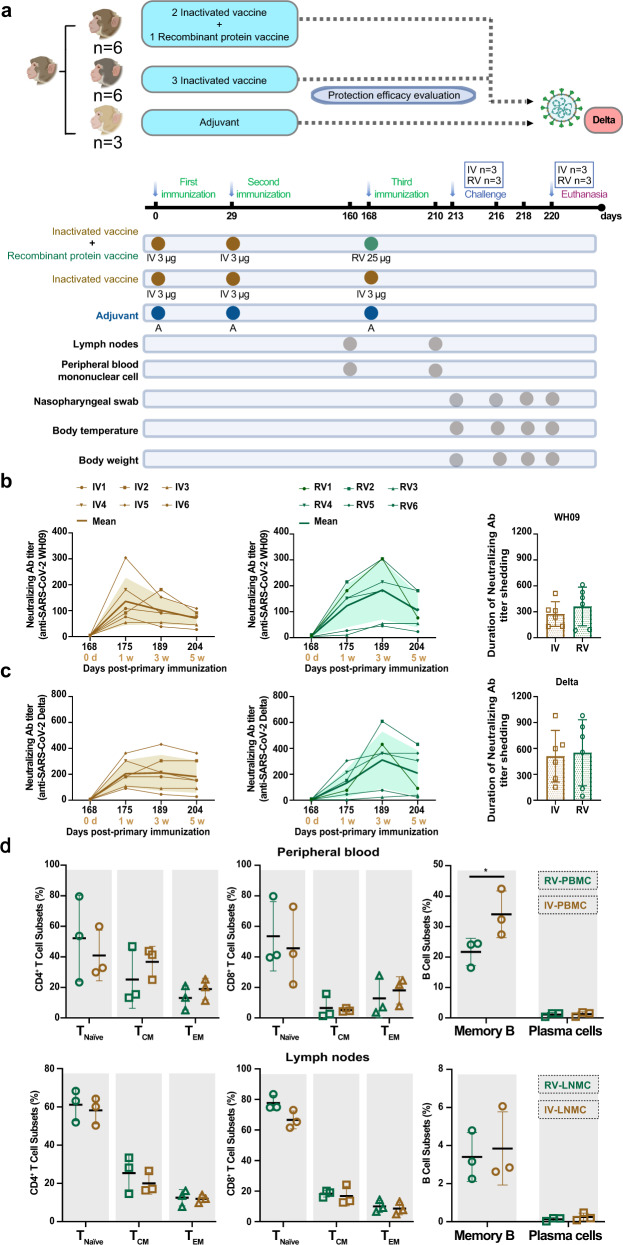
Sequential vaccination enhanced humoral and cellular immunity in *Rhesus macaques*. Fifteen adult Chinese-origin *Rhesus macaques* were enrolled in the study. At the outset of this experiment, 12 monkeys were immunized with inactivated vaccine on days 0, 29. Then the monkeys were randomly divided into two groups, named IV group (booster immunization with inactivated vaccine at day 168) and RV group (third dose with recombinant protein vaccine at the same time), respectively. Meanwhile, three monkeys were injected Al(OH)_3_ adjuvant at days 0, 29, 168 as control (named A group). **a** Experimental design and sample collection. **b** The titers of neutralizing antibodies against the WH09 strain in homologous or heterologous immunized monkeys. **c** The titers of neutralizing antibodies against the Delta variant in homologous or heterologous immunized monkeys post booster immunization. **d** Comparison of cellular immunity in peripheral blood (upper panel) as well as lymph nodes (lower panel) between homologous (IV group, *n* = *3*) and heterologous (RV group, *n* = *3*) prime-booster-immunized monkey at day 42 post the booster immunization (day 210 post-primary immunization). Significant differences are indicated with asterisks (*P < 0.05, **P < 0.01*;* Student’s *t* test)

The CoronaVac, a β-propiolactone-inactivated vaccine and an RBD-subunit vaccine against COVID-19, respectively, have been approved for emergency use.^15,16^ To investigate the effect of sequential immunization on neutralization antibody titers and their duration, adult Chinese-origin *Rhesus macaques* (4–7 kg, 4–7 years of age) were used in this study. The primates were divided into two groups - Group I, *n* = *12*, Coronavac and Group A, *n* = *3*, Al(OH)_3_ adjuvant. Animals from Group I were injected with 3 μg/dose of inactivated vaccine intramuscularly at day 0 and 29, while animals from Group A were injected with the adjuvant on the same days. After this, animals from Group I were divided into two groups and injected intramuscularly with either the 3rd homologous dose of inactivated vaccine (Group IV, *n* = *6*, 3 μg/dose, Coronavac) or heterologous recombinant protein vaccine (Group RV, *n* = *6*, 25 μg/dose) as a booster dose on day 168 after primary immunization. Meanwhile, the A group monkeys were injected Al(OH)_3_ adjuvant as control (Fig. 1a). Homologous booster vaccination resulted in higher NAb titers against the wild-type (WH09) strain. The NAb titers peaked 1 week after the booster dose administration and ranged from 1:54 to 1:304 in group IV. We observed that the NAbs titers for 5 weeks post the administration of the third dose. Heterologous booster immunization (prime-boost vaccination of inactivated vaccine followed by protein vaccine) also induced higher NAb titers against WH09 strain, peaking in 3 weeks post the third dose (range 1:45–1:304 in all group RV animals), which was maintained at the same level compared to that of the group IV animals (Fig. 1b). Likewise, we detected the level of NAbs against the Delta strain post booster vaccination. As shown in Fig. 1c, the NAbs titers against the Delta strain exhibited an enhancement in group IV animals at 1-week post third vaccination (range, 1:91–1:362). Similarly, the level of NAbs elicited against the Delta strain increased from 1-week post booster immunization and peaked at 3 weeks post heterologous booster immunization (range from 1:23 to 1:609 in all group RV animals). Moreover, the enhanced neutralizing antibodies levels against WH09 and Delta variants in the group IV animals appeared not significantly different compared to those in the RV group (*P* > 0.05) (Fig. 1b, c, right panel).

We collected samples of peripheral blood and lymph nodes at 160 days (7 days before the third dose) and 210 days (42 days post the third dose) to compare the immune responses before and after administration of the booster dose. The percentage of T cells and B cells in peripheral blood and lymph nodes from the RV group, including CD4^+^T subsets [naive CD4^+^ T cells (CD4^+^ T naive), central memory CD4^+^ T cells (CD4^+^ T_CM_), and effective memory CD4^+^ T cells (CD4^+^ T_EM_)], CD8^+^ T subsets (CD8^+^ T naive, CD8^+^ T_CM_, and CD8^+^ T_EM_), memory B cells and plasma cells on 42 days post the booster immunization showed no significant difference compared with those on 7 days before the third immunization (*P* > 0.05) (Supplementary Fig. S1a). The percentage of CD4^+^T subsets, CD8^+^ T subsets, and plasma cells in peripheral blood and lymph nodes were maintained at a similar level before and after the third immunization in group IV animals (*P* > 0.05). However, the percentage of memory B cells from group IV animals on day 210 showed an obvious increase when compared with those at day 160 (*P* < 0.05) (Supplementary Fig. S1b). Likewise, the percentage of memory B cells from PBMCs appears to increase significantly on day 210 compared with the same time point in the RV group (*P* < 0.05) (Fig. 1d). In addition, the percentage of T subsets and plasma cells showed no obvious difference between the homologous prime-boost regimens (group IV) and the heterologous-boost regimens (group RV) at 42 days post the booster vaccination (*P* > 0.05) (Fig. 1d). These results suggest that inactivated vaccine prime-boost regimens may be highly effective at recalling an immune response.

### Persistence of cross-neutralization antibody titers after sequential booster immunization

**Fig. 2:**
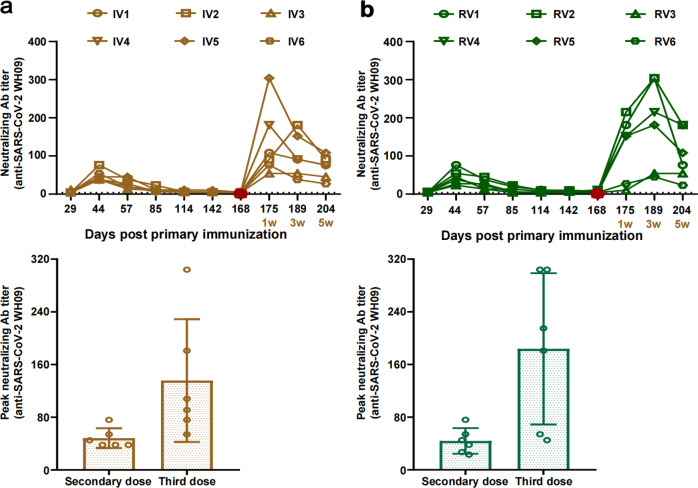
Longitudinal tracking of neutralizing antibody titers in *Rhesus macaques* with sequential vaccination. **a** Long-term observation of neutralizing antibodies titer for WH09 in IV monkeys. **b** Long-term observation of neutralizing antibodies titer for WH09 in RV monkeys. Significant differences are indicated with asterisks (*P < 0.05, **P < 0.01*;* Student’s *t* test)

Next, we monitored NAb titers against WH09 and Delta strains in immunized *Rhesus macaques* over a long period. Results showed that the NAbs against WH09 began to rise 29 days post-primary immunization, peaking at 44 days post-primary immunization (range 1:23–1:76, 14 days post the second dose), after which the titers gradually declined and were maintained at a lower level for 6 months (Fig. 2a). Priming immunization with inactivated vaccine followed by the third homologous dose (IV group) induced a 2.5-fold higher level of neutralizing antibodies against WH09 strain at 1-week post booster immunization [geometric mean titers (GMTs)=114] than that at 14 days post the secondary immunization. The GMTs of NAb against WH09 was 1:88 on 3 weeks and 1:64 on 5 weeks post the third dose (Fig. 2a). Next, we further estimated the duration of persistence of NAbs against WH09 and Delta, using blood samples taken at 10, 12, and 17 weeks after the third dose. The GMTs of NAb against WH09 were 1:24, 1:18, and 1:8 on 10, 12, and 17 weeks post homologous sequential immunization, and those against the Delta strain were 1:18, 1:16, and 1:10 during the same time. The levels of NAbs against WH09 and Delta showed a largely similar tendency to decline gradually (Supplementary Fig. S2). A heterologous sequential vaccination schedule involving prime-boost vaccination of inactivated vaccine followed by protein vaccine (RV group) increased the level of NAbs against WH09 and Delta. The peak geometric mean titers against WH09 post booster immunization were 1:144, which was 3.6-fold higher than 14 days post the secondary vaccination (Fig. 2b). Moreover, further analysis of the titers showed that the GMTs of NAbs against WH09 were 1:41, 1:34, and 1:19 and against Delta were 1:48, 1:38, and 1:26 at 10, 12 and 17 weeks post heterologous booster immunization, respectively (Supplementary Fig. S2). These results suggest that administration of a booster dose can significantly enhance the level of neutralizing antibodies against different variants of SARS-CoV-2 and that the NAbs titers can be sustained for at least 4 months.

### Sequential vaccination protected the monkeys from the infection of the SARS-CoV-2 Delta variant

**Fig. 3:**
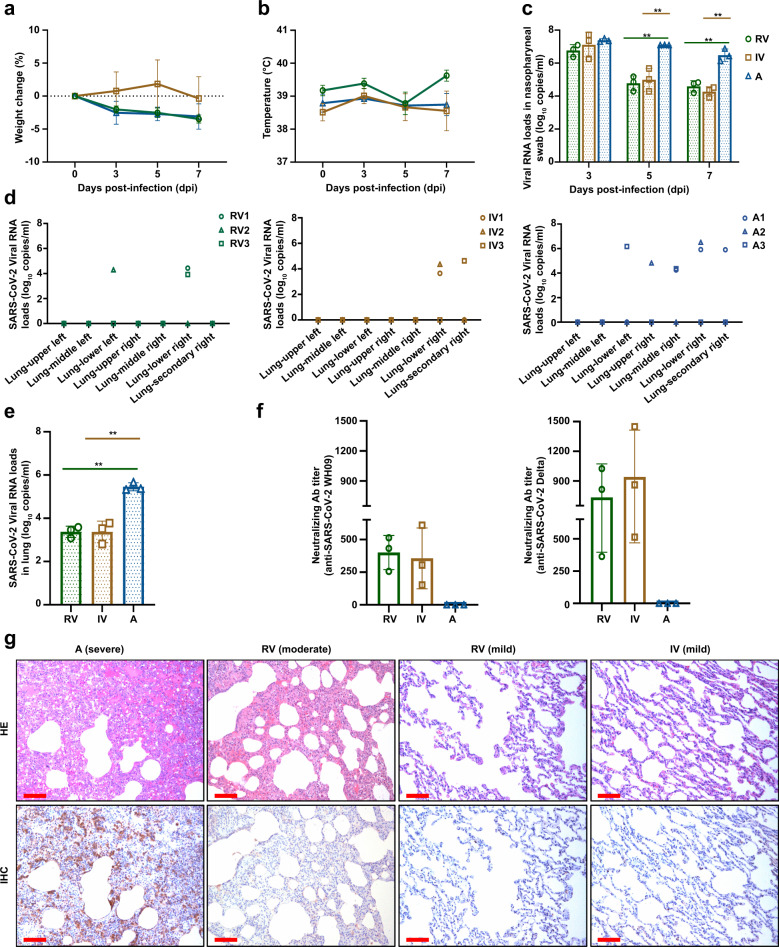
Sequential immunization protected *Rhesus macaques* against infection by the Delta variant. Three monkeys each belonging to the RV and IV groups were randomly selected and challenged intratracheally with the Delta variant (B.1.617.2) at 1 × 10^6^ TCID_50_. Similarly, the animals from the A group were infected with the Delta variant at the same dose as the control. **a**, **b** Monkeys were examined daily for changes in body weight and rectal temperature over the observation period after SARS-CoV-2 infection. **c** The SARS-CoV-2 RNA was detected by qRT-PCR in the nasopharyngeal swabs from all monkeys at the indicated time points. **d** Detection of viral RNA in different lung lobes from RV, IV, and A groups at 7 days post-infection. **e** The average viral loads in whole lungs in all monkeys. **f** Levels of neutralizing antibodies against the WH09 and Delta variants from each monkey at 7 dpi. **g** Lung tissues were collected from three groups of macaques at 7 dpi. The pathological changes were observed by hematoxylin and eosin (H&E) staining, and the viral antigens were detected by immunohistochemistry (IHC) against the spike protein of SARS-CoV-2 using serial sections. Red scale bar = 100 µm. Data sets are representative of three independent experiments

To investigate the effectiveness of the administration of a booster dose, animals were challenged with live viruses. Three primates were randomly selected from RV as well as IV groups and intratracheally challenged with the Delta strain of SARS-CoV-2 at 1 × 10^6^ TCID_50_ on day 213 (45 days after immunization with a booster dose). In addition, three monkeys from group A were infected with the SARS-CoV-2 Delta using the same dose; these animals served as a control group. Body weight, rectal temperature, nasopharyngeal swabs, virus distribution, pathological changes, immunocytes, and neutralizing antibodies were examined at the designated time points (Fig. 1a). The weight change fluctuated within normal ranges in the nine animals, and the rectal temperature was not elevated in any of the animals (Fig. 3a, b). To monitor the spread of the virus, nasopharyngeal swabs were collected at 3, 5, and 7 days post-infection (dpi) from RV, IV, and A groups and tested for their viral RNA loads. All control macaques showed excessive copies (>10^6.1^/ml) of viral genomic RNA in the pharynx at day 3–7 dpi and median viral loads (10^4.0^/ml–10^6.5^/ml) in lungs, but developing severe interstitial pneumonia (Fig. 3c–g).

The peak viral load in nasopharyngeal swabs from all vaccinated macaques was detected at 3 dpi, followed by a substantial decline over time. The average viral RNA copies in nasopharyngeal swabs of RV and IV animals were 10^4.91^ and 10^5.29^ at 5 dpi, respectively, showing an approximately 100-fold reduction compared to those at 3 dpi. However, macaques from the control group sustained robust viral replication in the nasopharynx at 3–7 dpi (Fig. 3c). Similarly, the average viral loads in nasopharyngeal swabs of the RV and IV groups decreased further with genomic RNA copies of 10^4.67^ and 10^4.33^ at 7 dpi, respectively. These viral loads were ~100-fold lower compared to those of the control animals (Fig. 3c). At 7 dpi, monkeys from these three groups were euthanized for viral RNA detection and histopathological observation. Low-level SARS-CoV-2 RNA loads were detected in the lower lobes (10^3.42^ RNA copies/mL on average in the intact lung) in the group RV animals. Low-level viral loads were detected in the right lobes, and no viral RNA in the left lobes was observed from group IV animals, the average RNA copies in the whole lungs of group IV animals was 10^3.52^/ml (Fig. 3d). The average viral load in the entire lung from RV and IV animals was significantly reduced compared with those in the control group (*P* < 0.01) (Fig. 3e). Next, the neutralizing antibody titers were also assessed on 7 dpi. The authentic neutralization assays showed that the NAb titers against WH09 rose equally in both RV (GMT = 384) and IV groups (GMT = 304). In addition, the NAb titers against the Delta strain were significantly increased in the RV group (range, 1:362–1:1024) and IV group (range, 1:512–1:1448), which were approximately fourfold higher than those before infection (Fig. 3f). It is of importance to note that none of neutralizing antibodies neither against WH09 nor Delta was detected in the control group macaques on 7 dpi (Fig. 3f), since it generally takes 2–3 weeks to elicit NAbs after primary infection/vaccination. These indicate that selective and expeditious recall of humoral responses can be elicited via the Delta or other variants of infection. In addition, we further examined the histological lesions in the lungs. Non-vaccinated animals infected with SARS-CoV-2 Delta variant developed diffuse alveolar damage and severe interstitial pneumonia characterized by multifocal to coalescing widened alveolar interstitium, extensive edema, massive infiltration of inflammatory cells, and desquamated cells filled in the alveolar lumina, moderate pneumocyte proliferation, mild to moderate perivasculitis, and vasculitis (Fig. 3g). As opposed to this, all (3/3) the vaccinated macaques belonging to group IV were largely protected against the SARS-CoV-2 Delta variant exhibiting almost normal to focal and mild pathological changes (Fig. 3g). Animals belonging to the RV group were partially protected against the SARS-CoV-2 Delta variant showing mild (2/3) and moderate (1/3) lesions. Importantly, no thrombosis, vasculitis, or perivasculitis was observed in the lungs from both the IV and RV groups, and no significant pathological changes were also detected in other tissues. Immunohistochemical staining was used to further confirm the observations garnered through HE staining. The viral protein was expressed in the bronchiolar epithelial cells, alveolar epithelial cells, and alveolar macrophages in the Delta variant challenged control group A, while viral proteins were rarely observed when stained for in the RV and IV groups (Fig. 3g). These results suggest that an increased titer of neutralizing antibodies was induced against SARS-CoV-2 by homologous and heterologous boosters after sequential immunization, which protected the non-human primates against the Delta variant infection. Furthermore, the analysis of NAbs elicited by a homologous inactivated vaccine booster dose revealed the ability of the vaccination regimen to confer broad-spectrum protection against variants. Such a strategy could also expeditiously recall responses of immune cells against SARS-CoV-2 when compared to a heterologous booster dose.

### Sequential vaccination protected the monkeys from the infection of SARS-CoV-2 Omicron variant

**Fig. 4:**
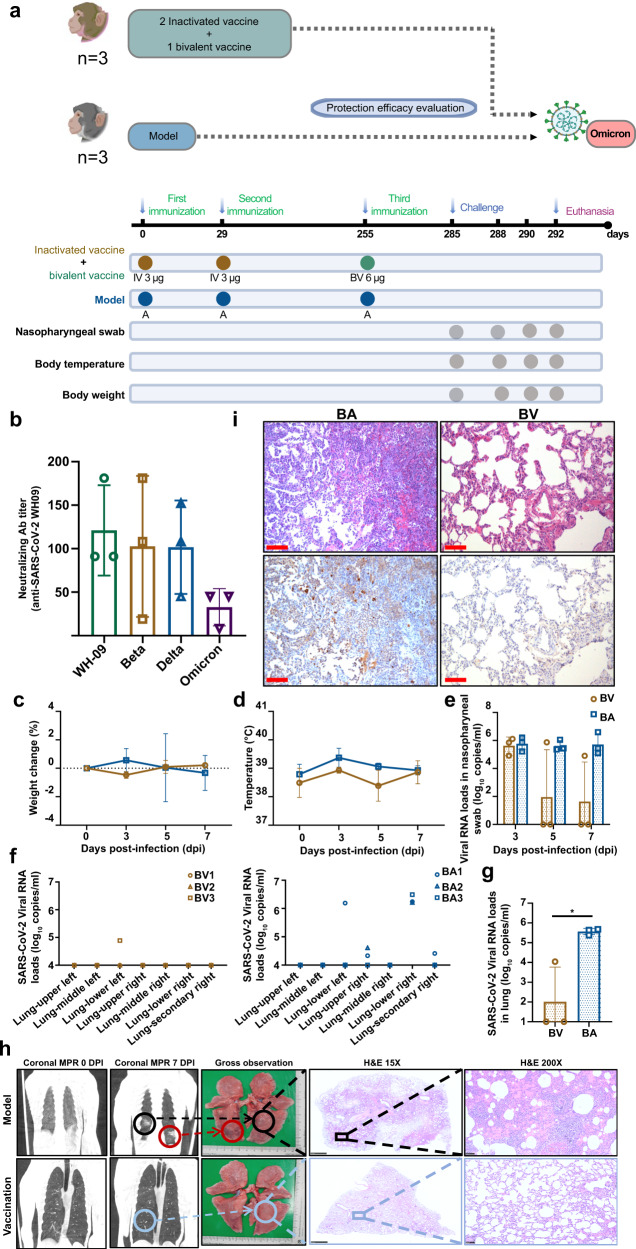
Sequential vaccination protected monkeys from Omicron infection. Six adult Chinese-origin *Rhesus macaques* were enrolled in this study. At the outset of this experiment, three monkeys were immunized with inactivated vaccine at days 0, 29 and re-immunized with the inactivated bivalent vaccine at day 255 (named BV group). The other three monkeys were injected Al(OH)_3_ adjuvant at day 0, 29, 255 as control (named BA group). All the BV and BA monkeys were challenged intratracheally with the Omicron variant at 1 × 10^6^ TCID_50_. **a** Experimental design and sample collection. **b** The neutralizing antibody titers against WH09, Beta, Delta, and Omicron were detected in animals re-immunized with the bivalent vaccine. **c**, **d** Monkeys were examined daily for changes in body weight and rectal temperature over the observation period after SARS-CoV-2 infection. **e** The SARS-CoV-2 RNA was detected by qRT-PCR in the nasopharyngeal swabs from all monkeys at the indicated time points. **f** Detection of viral RNA in different lung lobes from BV and BA groups at 7 days infection post-infection. **g** The average viral loads in whole lungs in all monkeys. **h** Observation of the results of CT imaging (MPR, multi-planar reconstruction), gross lesions, and microscopic examination and comparison between the animal model and the vaccination group. Black circle, lesions in the right lower lobe of the lung in the model group; red circle, lesions in the left lower lobe of the lung in the model group; blue circle, the same location in the vaccination group in the right lower lobe of the lung while showing focal and mild changes. Data are representative of three independent experiments. **i** Lung tissues were collected from three groups of macaques at 7 dpi. The pathological changes were observed by hematoxylin and eosin (H&E) staining, and the viral antigens were detected by immunohistochemistry (IHC) against the spike protein of SARS-CoV-2 using serial sections. Red scale bar = 100 µm. Data (**i**) are representative of three independent experiments

We have confirmed that sequential immunization seems to confer improved protective efficacy against Delta challenge, we set out to further optimize the vaccination regime against Omicron via sequential immunization with a booster dose using a bivalent vaccine (inactivated β + δ). To verify this, three macaques (BV1-BV3) were firstly immunized with 3 μg/dose of inactivated vaccine (Coronavac, wild-type strain) at day 0, and 29 and then vaccinated with a bivalent booster dose (6 μg/dose, inactivated β + δ) at day 255. In parallel, the BA1-BA3 monkeys were administrated with Al(OH)_3_ adjuvant at days 0, 29, and 255 as control (Fig. 4a). Blood samples were collected at 30 days post booster vaccination to evaluate the neutralizing antibody titers against WH09, Beta, Delta, and Omicron variants (Fig. 4b). As shown in Fig. 4b, the geometric mean NAb titers against the Omicron variant were 1:25.3, which were 4.5-fold lower than those against the ancestral virus (WH09, 1:114) on 30 days post booster immunization. Interestingly, the geometric mean NAb titers against Beta and Delta reached up to 1:70, comparable to those against WH09 (Fig. 4b). Subsequently, all six monkeys were intratracheally challenged with SARS-CoV-2 Omicron at 1 × 10^6^ TCID_50_ on days 285 (30 days after immunization with a booster dose). The weight change and the rectal temperature fluctuated within normal ranges in these monkeys (Fig. 4c, d). The viral loads in nasopharyngeal swabs from the control group (BA group) monitored from 3 dpi to 7 dpi, ranged from 10^5.10^ to 10^6.59^copies/ml, however, the viruses in pharyngeal specimens from 2/3 BV-monkeys could be completely cleared at days 5 and 7 post-infection (Fig. 4e). All monkeys from each group were euthanized and necropsied at 7 dpi. Individual lobes of the lung were collected for estimating the viral loads. As shown in Fig. 4f, high levels of viral RNAs ranging from 10^4.33^ to 10^6.49^copies/ml were detected in the majority of the lobes of lungs in BA monkeys. In contrast, only one monkey from the BV group showed the presence of viruses in the lower lung with lower viral copies (10^4.89^/ml). The averaged viral load in the lung of BV animals was significantly decreased compared to those in the control group (*P* < 0.05) (Fig. 4g). There were obvious pathological damages in the lungs of control animals at 7 dpi, including prominent ground-glass opacities and patchy lesions in the lower bilateral lobes of the lung (Fig. 4h). By contrast, macaques in the vaccination group displayed normal morphology of the lung. Consistent with the CT results, gross observation of the control macaques revealed the presence of lesions on the dorsal side of lower bilateral lobes of the lungs, in which scattered dark-reddish-purple areas were distinctly noted. During the histopathological examination of samples, monkeys infected with SARS-CoV-2 Omicron variant developed diffuse alveolar damage similar to those observed for the Delta variant, while all the vaccinated macaques (3/3) were largely protected against SARS-CoV-2 Omicron variant, exhibiting almost normal to focal and mild histological lesions without any thrombosis, vasculitis, or perivasculitis. In addition, no significant abnormalities were observed in other tissues. Results of the immunohistochemical staining were consistent with the H&E observations (Fig. 4i). These results demonstrate the protective effect of sequential immunization of a bivalent vaccine against the Omicron variant in monkeys, suggesting that sequential immunization could protect people from infections caused by Omicron.

## Discussion

SARS-CoV-2 seems to evolve continuously with variants produced by errors introduced during replication getting selected under varying selection pressures. Some of the variants may escape the majority of SARS-CoV-2 neutralizing antibodies induced by current vaccines, which has triggered a race to accelerate booster vaccination programs. *Rhesus macaque* has the most similar immune response profiles to humans. In this paper, the 2-dose inactivated vaccine immunized monkeys were re-immunized with the inactivated vaccine (Coronavac, IV group) or an RBD-subunit vaccine (RV group), the NAbs titers to wh09 appear an obvious increase in both IV and RV group post the booster vaccination, which exceeds twofold than that the peak titer enhanced post the secondary vaccination, and the increased titer level showed no significant difference between IV and RV group (Figs. 1b, c and 2a). It is noteworthy that the peak neutralizing antibody titers enhanced by inactivated vaccine booster immunization (IV group) appeared on 1 week, however, the peak titer in the RV group was detected 3 weeks post protein vaccine boosting vaccination, which indicates that homologous sequential immunization can activate immune response faster than heterologous booster vaccination strategy. Similar results have been reported for studies on inactivated vaccine and mRNA vaccine booster immunization regimens that markedly increased the levels of B- and T-cell response.^7,11,17,18^ However, the actual percentage of change in immune cells with RBD recombinant vaccine booster strategy has not been reported. In our study, the percentage of memory B cells in peripheral blood from IV monkeys post the third dose was remarkably enhanced compared with that in the RV group and significantly increased compared with the point before the third dose (Fig. 1d), suggesting that the 2-dose inactivated vaccine immunization equipped the individual with immune memory. Although the third dose of inactivated vaccines rapidly enhanced memory response data on antigen-specific B and T cells population after the booster vaccination is lacking, and should be further investigated and compared in both group IV and RV animals. In addition, further studies are needed to confirm the relevance between memory B responses and protection against SARS-CoV-2 variants. Inactivated vaccine prime-booster immunization strategy can enhance the cross-neutralizing antibodies, recall immune memory, and quickly exploit the advantages of protection within a short period. These results illustrated that inactivated vaccine booster vaccinated regime will play an important role in epidemic prevention.

At present, the Omicron variant has been reported to have a reproduction number (R0) in the range of 2–5.^19–21^ Whether the Omicron variant of SARS-CoV-2 can escape the existing herd immunity built by massive vaccinations in a large scale the human population is a crucial question. Preliminary studies suggested that Omicron with an unprecedented number of mutations in its spike presumably confers greater resistance to neutralizing antibodies elicited by mRNA, DNA, and inactivated vaccines.^22,23^ Our more recent study demonstrated the presence of a subset of antibodies with broad neutralizing activities against all circulating VOCs, including Omicron in memory B-derived antibody repertoire from the 3-dose vaccinees, which suggests a possibility that selective and expeditious recall of humoral responses might be elicited via the Omicron infection, conferring to secondary protection directed by memory etched in the immune system.^7^ In this paper, we detect the effectiveness of bivalent inactivated vaccine (inactivated β + δ) booster immunization to different stains, found that bivalent vaccine sequential immunization appears an obvious validity to WH09, Delta as well as Omicron variants in monkeys. In addition, only 1/3 of BV-monkeys (the bivalent vaccine booster-immunized animals) were detected the viral loads from lung tissues, which confined to only one lung lobe, and the load viruses were completely cleared in other lungs of BV-monkeys. All the third dose booster-immunized monkeys (3/3 BV-monkeys) exhibited almost normal to focal and mild histological lesions without thrombosis, vasculitis, or perivasculitis, indicating that repeated immunizations of inactivated vaccine can provide nearly complete protection in macaques without observable vaccine/antibody-mediated enhancement of infection. More recently, a recombinant virus called “Deltacron” has been reported due to the co-circulation of Omicron and Delta. Although most mutations in Deltacron’s spike protein derive from Omicron, the impact of the recombination on viral transmissibility and the ability to escape neutralizing antibodies elicited by vaccination is unclear.^24^ Our results confirmed the effectiveness of the bivalent inactivated vaccine sequential immunization strategy against Omicron in vivo and in *Rhesus macaques*, which will rationally guide vaccination regimes against COVID-19.

Taken together, homologous inactivated vaccine prime-boost regimens appear to induce long-lasting neutralizing antibodies titers and higher levels of the immune response. Notably, these responses seem to be effective against a broader range of strains. Therefore, incorporation of a third dose of inactivated vaccine as part of a complete immunization program, or employing the polyvalent inactivated vaccine strategy, could provide more durable protection against circulating variants. The bivalent vaccine be highly effective against variants mutated, including Omicron, which indicates that the bivalent vaccine can act as an emergency preventive strategy to combat with future variants.

## Materials and methods

### Ethics statement

All animal procedures were approved by the Institutional Animal Care and Use Committee of the Institute of Laboratory Animal Science, Peking Union Medical College (ILAS, PUMC) (No. DW21007). All experiments were performed in an animal biosafety level 3 (ABSL3) facility with high-efficiency particulate air (HEPA)-filtered isolators.

### Virus

The SARS-CoV-2 virus is designated as SARS-CoV-2/human/CHN/Delta-1/2021 (GenBank: OM061695.1) and SARS-CoV-2/human/CHN/Omicron-1/2021 (Genbank: OM095411.1) were provided by ILAS, PUMC, China. To identify the stocks of the virus, the plaque purified viral isolate was amplified as described previously.^25–27^ Titers for SARS-CoV-2 were determined using a median TCID_50_ assay.

### Immunization program and challenge assay of *Rhesus macaques* (Part I)

*Rhesus macaques* (4–7 years old) were divided into three groups and injected intramuscularly with 3 μg/dose inactivated vaccine (named I, *n* = *12*, Coronavac) and Al(OH)_3_ adjuvant (named A, *n* = *3*). All animals from the I group were immunized with two doses of the inactivated vaccine (at days 0 and 29). Then the monkeys were divided into two groups and injected intramuscularly with the 3rd dose of inactivated vaccine (IV, *n* = *6*, 3 μg/dose, Coronavac) or recombinant protein vaccine (RV, *n* = *6*, 25 μg/dose) at day 168 after primary immunization. Meanwhile, the A group monkeys were injected Al(OH)_3_ adjuvant as control. Animals (RV1-3, IV1-3, and A1-3) were challenged with 10^6^ TCID_50_/ml SARS-CoV-2 (Delta variant) virus by intratracheal routes at day 213 after primary immunization (45 days after third immunization). On days 3, 5, and 7 dpi, the nasopharyngeal swabs were collected. Macaques were euthanized and lung tissues were collected at 7 dpi and used for RT-PCR and histopathological assays. Blood samples were collected on 1, 3, 5, 10, 12, and 17 weeks post third immunization, and 7 days post-infection for neutralizing antibody test of SARS-CoV-2. The study design and longitudinal sampling schedule are shown in Fig. 1a.

### Immunization program and challenge assay of *Rhesus macaques* (Part II)

Three *Rhesus macaques* (4–7 years old) were injected intramuscularly with 3 μg/dose inactivated vaccine (Coronavac) on 0, 29 days, and then injected with bivalent vaccine (Group BV, 6 μg/dose, Coronavac β + δ) after 7 months (BV, *n* = *3*). In addition, three monkeys were injected with Al(OH)_3_ adjuvant at days 0, 29, and 255, which served as the control group (BA, *n* = *3*).

All animals were infected with 10^6^ TCID_50_/ml SARS-CoV-2 (Omicron) virus by intratracheal routes on day 285 after primary immunization (30 days after third immunization). On days 3, 5, and 7 dpi, the nasopharyngeal swabs were collected. Macaques were euthanized and lung tissues were collected at 7 dpi for RT-PCR and histopathological assays. Blood samples were collected on day 30 post third immunization for neutralizing antibody test of SARS-CoV-2. The study design and longitudinal sampling schedule are shown in Fig. 4a.

### Authentic neutralization assay

Serum samples were tested for the presence of neutralizing antibodies by cytopathic effect (CPE). Briefly, the sera from monkeys were heat-inactivated at 56 °C for 30 min. After inactivation, twofold serially diluted sera were incubated with 100 TCID_50_ SARS-CoV-2 for 1 h at 37 °C and added to Vero-E6 cells in a 96-well-plate. Cells were cultured for 3–4 days to observe CPE, and the serum dilution in which 50% of the cells were protected against infection was calculated.

### Quantification RT-PCR

Total RNA was extracted and reverse transcription was performed as described previously.^25^ Briefly, qRT-PCR was carried out using the following cycling protocol and primers: 50 °C for 2 min, then 95 °C for 2 min, followed by 40 cycles of 95 °C for 15 s and 60 °C for 30 s, and final incubations at 95 °C for 15 s, 60 °C for 1 min, and 95 °C for 45 s. The following primers were used to detect SARS-CoV-2: SARS-CoV-2: forward primer, 5′-TCGTTTCGGAAGAGACAGGT-3′; reverse primer, 5′-GCGCAGTAAGGATGGCTAGT-3′.

### Flow cytometry

Lymph node biopsies were performed and then treated with 5 mM EDTA and 60 U/ml collagenases. Lymph node mononuclear cells (LNMCs) were enriched for lymphocytes using Percoll density-gradient centrifugation, and peripheral blood mononuclear cells (PBMCs) were isolated using conventional Ficoll-Hypaque density-gradient centrifugation (GE Healthcare, Uppsala, Swed en). Polychromatic flow cytometry was performed to analyze CD4^+^ or CD8^+^ T lymphocyte subsets (CD3^+^ CD4^+^/CD8^+^CCR7^+^CD45RA^+^ for naive T cells, CD3^+^CD4^+^/CD8^+^CCR7^+^CD45RA^−^ for central memory T cells, CD3^+^CD4^+^/CD8^+^CCR7^−^CD45RA^−^ for effective memory T cells), memory B cells (CD3^−^CD20^+^CD27^+^) and plasma cells (CD3^−^CD20^+^CD43^+^). T lymphocyte subsets from LNMCs or PBMCs were stained with the CD3-BV605 (SP34-2, BD Biosciences, San Jose, CA, 562994), CD4 BV785 (OKT-4, Biolegend, 317442), CD8-PE (RPAT8, BD Biosciences, 557086), CCR7-BV421 (G043H7, Biolegend, 352208), and CD45RA-APC (5H9, BD Biosciences, 561210) monoclonal antibodies. B lymphocyte subsets from LNMCs or PBMCs were stained with CD3-PE (SP34-2, BD Biosciences, 552127), CD20-BV785 (2H7, Biolegend, 302356), CD-27 BV711 (O323, Biolegend, 302834), and CD43-APC (CD43-10G, Biolegend, 343206) monoclonal antibodies. The cells were resuspended in 1% paraformaldehyde and subjected to flow cytometry analyses within 24 h. All samples were analyzed using flow cytometry (FACSAria; BD, CA).

### Hematoxylin and eosin (H&E) staining and immunohistochemistry (IHC)

A 10% buffered formalin solution was prepared to fix all the collected tissues and organs. Paraffin sections (3–4 mm in thickness) were cut following established procedures. All the lung sections were stained with hematoxylin and eosin. The histopathological observation was carefully recorded by observing slides using an Olympus microscope. For IHC staining, to identify the expression of SARS-CoV-2 S protein (GTX635654, 1:200; GeneTex, Irvine, CA), dehydrated paraffin sections (3–4 µm in thickness) were treated with an antigen retrieval kit (AR0022; Boster Bio, Pleasanton, CA) and quenched for endogenous peroxidases in three percent H_2_O_2_ in methanol for 10 min. After blocking in 1% normal goat serum for 1 h at 37 °C, the sections were stained with anti-SARS-CoV-2 rabbit monoclonal antibody at 4 °C overnight, followed by incubation with a horseradish peroxidase HRP-labeled goat anti-rabbit IgG secondary antibody (ZDR-5306, 1:200; ZSGB Bio) for 1 h at 37 °C. Finally, the sections were visualized by incubation with 3,3′-diaminobenzidine tetrahydrochloride (DAB) and viewed carefully by an Olympus microscope.

### CT

Macaques were anesthetized with 2% isoflurane. CT imaging was conducted by using an IRIS XL-260 CT scanner (Inviscan SAS, Strasbourg, France) with the following settings: tube voltage of 80 kV, tube current of 900 µA, exposure time of 50 ms, and 1280 projections over 360-degree rotation. Images were reconstructed by Feldkamp filtered projection back-projection algorithm with 150 µm isotropic voxel size. Images acquired from the scanner were viewed and analyzed in OsiriX MD software.

### Statistical analysis

All data were analyzed with GraphPad Prism 8.0 software (GraphPad Software, Inc). Comparisons among groups were performed by the two-tailed unpaired Student’s *t* test. The level of statistical significance was determined as *P < 0.05, **P < 0.01.

## Supplementary information

Revised_Sigtrans_Supplementary_Materials_Word_SIGTRANS-06089R

## Data Availability

All data needed to evaluate the conclusions in the paper are present in the paper and/or the supplementary materials.

## References

[1] Luo, C. H. et al. Infection with the SARS-CoV-2 delta variant is associated with higher infectious virus loads compared to the alpha variant in both unvaccinated and vaccinated individuals. *Clin. infect.dis*. https://www.medrxiv.org/content/10.1101/2021.08.15.21262077v1 (2021). 10.1093/cid/ciab986 PMC8903351 34922338

[2] Cameroni E (2022). Broadly neutralizing antibodies overcome SARS-CoV-2 Omicron antigenic shift. Nature.

[3] Cui Z (2022). Structural and functional characterizations of infectivity and immune evasion of SARS-CoV-2 Omicron. Cell.

[4] Shiehzadegan S, Alaghemand N, Fox M, Venketaraman V (2021). Analysis of the delta variant B.1.617.2 COVID-19. Clin. Pract..

[5] Farinholt T (2021). Broadly neutralizing antibodies overcome SARS-CoV-2 Omicron antigenic shift. BMC. Med.

[6] Greaney AJ (2021). Comprehensive mapping of mutations in the SARS-CoV-2 receptor-binding domain that affect recognition by polyclonal human plasma antibodies. Cell Host Microbe.

[7] Wang K (2022). Memory B cell repertoire from triple vaccinees against diverse SARS-CoV-2 variants. Nature.

[8] Edara VV (2022). mRNA-1273 and BNT162b2 mRNA vaccines have reduced neutralizing activity against the SARS-CoV-2 omicron variant. Cell. Rep. Med.

[9] Tseng, H. F. et al. Effectiveness of mRNA-1273 against SARS-CoV-2 omicron and delta variants. *Nat. Med.* (2022). 10.1038/s41591-022-01753-y PMC9117141 35189624

[10] Ai J (2022). Omicron variant showed lower neutralizing sensitivity than other SARS-CoV-2 variants to immune sera elicited by vaccines after boost. Emerg. Microbes Infect..

[11] Zeng G (2021). Immunogenicity and safety of a third dose of CoronaVac, and immune persistence of a two-dose schedule, in healthy adults: interim results from two single-centre, double-blind, randomised, placebo-controlled phase 2 clinical trials. Lancet. Infect. Dis..

[12] Wang, K. et al. A third dose of inactivated vaccine augments the potency, breadth, and duration of anamnestic responses against SARS-CoV-2. Preprint at https://www.medrxiv.org/content/10.1101/2021.09.02.21261735v1 (2021).10.1093/procel/pwae03338801319

[13] Zhang J (2021). Boosting with heterologous vaccines effectively improves protective immune responses of the inactivated SARS-CoV-2 vaccine. Emerg. Microbes Infect..

[14] He Q (2021). Heterologous prime-boost: breaking the protective immune response bottleneck of COVID-19 vaccine candidates. Emerg. Microbes Infect..

[15] Gao Q (2020). Development of an inactivated vaccine candidate for SARS-CoV-2. Science.

[16] Yang S (2021). Safety and immunogenicity of a recombinant tandem-repeat dimeric RBD-based protein subunit vaccine (ZF2001) against COVID-19 in adults: two randomised, double-blind, placebo-controlled, phase 1 and 2 trials. Lancet Infect. Dis..

[17] Liu Y (2022). Robust induction of B cell and T cell responses by a third dose of inactivated SARS-CoV-2 vaccine. Cell Discov..

[18] Zuo, F. et al. Heterologous immunization with inactivated vaccine followed by mRNA booster elicits strong humoral and cellular immune responses against the SARS-CoV-2 Omicron variant. Preprint at https://www.medrxiv.org/content/10.1101/2022.01.04.22268755v2 (2022). 10.1038/s41467-022-30340-5 PMC9106736 35562366

[19] Holgersen EM (2021). Transcriptome-wide off-target effects of steric-blocking oligonucleotides. Nucleic Acid Therapeut..

[20] Callaway E (2021). Heavily mutated Omicron variant puts scientists on alert. Nature.

[21] Ito K, Piantham C, Nishiura H (2021). Relative instantaneous reproduction number of Omicron SARS-CoV-2 variant with respect to the Delta variant in Denmark. J. Med. Virol..

[22] Cele S (2022). micron extensively but incompletely escapes Pfizer BNT162b2 neutralization. Nature.

[23] Lu, L. et al. Neutralization of SARS-CoV-2 Omicron variant by sera from BNT162b2 or Coronavac vaccine recipients. *Clin. Infect. Dis.* (2021). 10.1093/cid/ciab1041 PMC8754807 34915551

[24] Colson, P. et al. Culture and identification of a Deltamicron SARS-CoV-2 in a three cases cluster in southern France. Preprint at https://www.medrxiv.org/content/10.1101/2022.03.03.22271812v2 (2022). 10.1002/jmv.27789 PMC9088576 35467028

[25] Bao L (2020). The pathogenicity of SARS-CoV-2 in hACE2 transgenic mice. Nature.

[26] Deng W (2020). Primary exposure to SARS-CoV-2 protects against reinfection in *Rhesus macaques*. Science.

[27] Deng W (2020). Ocular conjunctival inoculation of SARS-CoV-2 can cause mild COVID-19 in *Rhesus macaques*. Nat. Commun..

