# Supplementary material for: Sequential immunizations confer cross-protection against variants of SARS-CoV-2, including Omicron in Rhesus macaques
Source: Signal Transduct Target Ther. 2022 Apr 18;7:124. doi: 10.1038/s41392-022-00979-z (PMC9014776; doi:10.1038/s41392-022-00979-z)
Supplement: Supplementary file 1 — Revised_Sigtrans_Supplementary_Materials_Word_SIGTRANS-06089R [file 41392_2022_979_MOESM1_ESM.pdf]

Supplementary Materials for

Sequential immunizations confer cross-protection against variants of SARS-CoV-2, including Omicron in *Rhesus macaques*

Wei Deng[1,2†], Qi Lv[1,2†], Fengdi Li[1,2†], Jiangning Liu[1,2†], Zhiqi Song[1,2†], Feifei Qi[1,2], Qiang Wei[1,2], Pin Yu[1,2], Mingya Liu[1,2], Shasha Zhou[1,2], Yaqing Zhang[1,2], Hong Gao[1,2], Nan Wang[3], Zijing Jia[3],Kai Gao[1,2], Jiayi Liu[4], Chong Xiao[1,2], Haiquan Shang[1,2], Xiangxi Wang[3*],Linlin Bao[1,2*], Chuan Qin[1,2*]

Correspondence to: Chuan Qin (qinchuan@pumc.edu.cn; Fax +086-10-67710812); Linlin Bao (Email: bllmsl@aliyun.com);Xiangxi Wang (Email: xiangxi@ibp.ac.cn)

These authors contributed equally: Wei Deng, Qi Lv, Fengdi Li, Jiangning Liu, Zhiqi Song

This PDF file includes:

Figures. S1 to S2

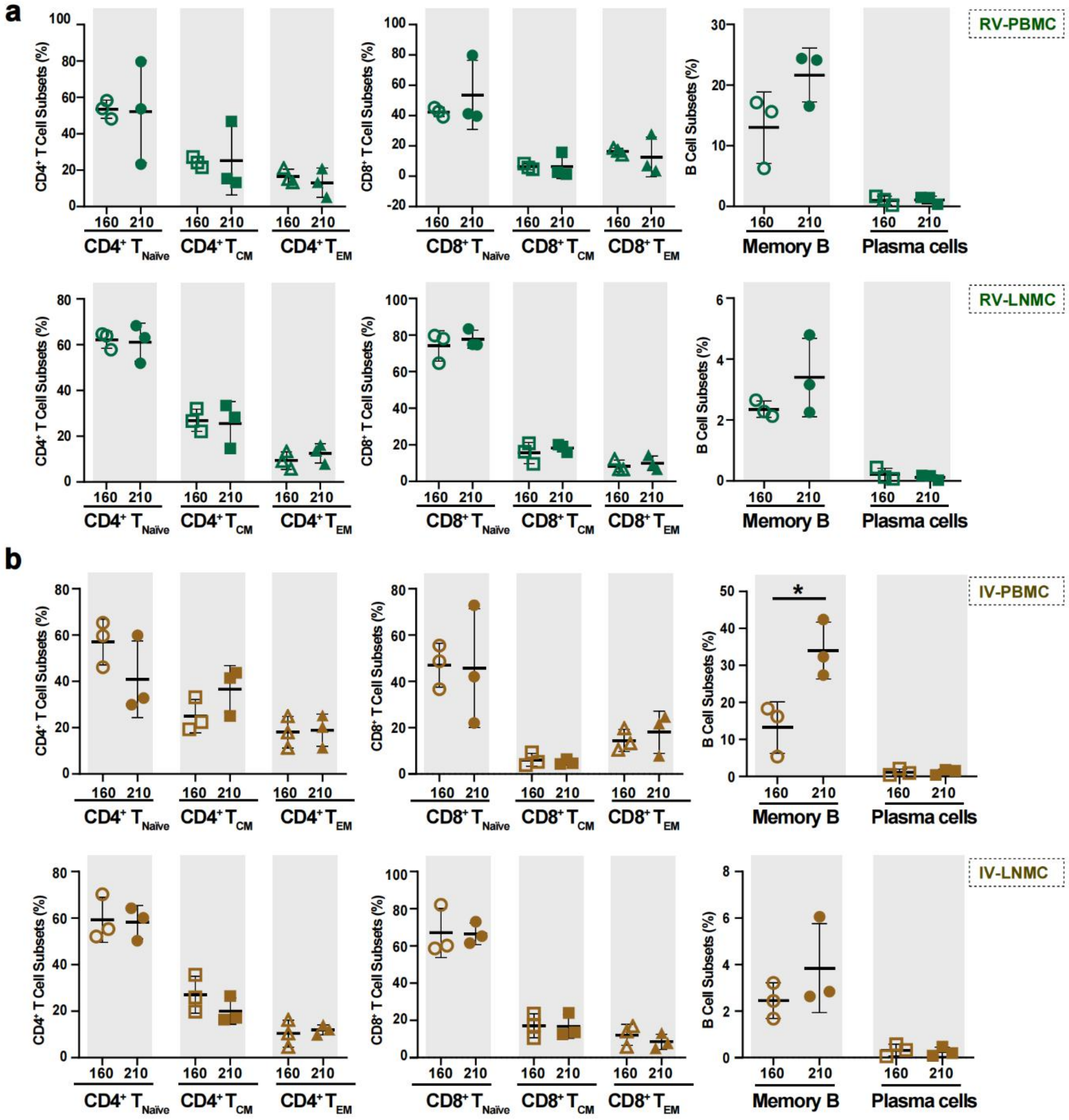

Figure. S1.

Comparison of cellular immunity between before and after the booster immunization in rhesus macaques with sequential vaccination. (a) Comparison of cellular immunity between 160 days (7 days before the third dose) and 210 days (42 days post the third dose) in heterologous prime-booster immunized monkey (RV group). (b) Comparison of cellular immunity between 160 days and 210 days in homologous prime-booster immunized monkey (IV group). Significant differences are indicated with asterisks (*$p < 0.05$, **$p < 0.01$; Student's t-test).

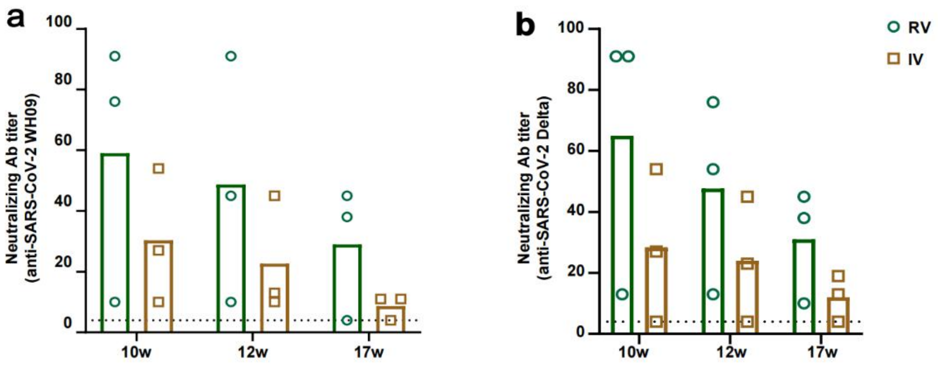

Figure. S2.

Longitudinal tracking of neutralizing antibody titers in rhesus macaques with sequential vaccination post the third dose. (a) The level of NAbs against the WH09 variant was detected at the indicated time points post the third dose. (b) The level of NAbs against the Delta variant was detected at the indicated time points post the third dose. Significant differences are indicated with asterisks (*p ＜ 0.05, **p ＜ 0.01; Student’ s t-test).
